# Molecular prevalence, characterization and associated risk factors of *Anaplasma* spp. and *Theileria* spp. in small ruminants in Northern Pakistan

**DOI:** 10.1051/parasite/2020075

**Published:** 2021-01-08

**Authors:** Sadaf Niaz, Zia Ur Rahman, Ijaz Ali, Raquel Cossío-Bayúgar, Itzel Amaro-Estrada, Abdullah D. Alanazi, Irfan Khattak, Jehan Zeb, Nasreen Nasreen, Adil Khan

**Affiliations:** 1 Department of Zoology, Abdul Wali Khan University Mardan Toru Road, Sheikh Maltoon Town 23200 Mardan Pakistan; 2 Centro Nacional de Investigación Disciplinaria en Salud Animal e Inocuidad, Instituto Nacional de Investigaciones Forestales Agrícolas y Pecuarias INIFAP, Carr. Fed. Cuernavaca-Cuautla No. 8534 Jiutepec 62550 Morelos México; 3 Department of Biological Sciences, Faculty of Science and Humanities, Shaqra University P.O. Box 1040 11911 Ad-Dawadimi Saudi Arabia

**Keywords:** *Anaplasma ovis*, *Theileria annulata*, *Theileria ovis*, *Theileria lestoquardi*, Sheep, Goat, Phylogeny, Risk factors, Pakistan

## Abstract

This study was conducted in four districts (Malakand, Swat, Bajaur and Shangla) of Northern Pakistan to investigate the prevalence, associated risk factors and phylogenetic analyses of *Theileria* and *Anaplasma* species in small ruminants. A total of 800 blood samples, 200 from each district, were collected from apparently healthy animals. PCR assays were performed using generic primers for *Anaplasma* spp. and *Theileria* spp. as well as species specific primers for *A. ovis* and *T. ovis*. Overall infection prevalence was 361/800 (45.1%). *Theileria* spp. infection prevalence (187/800, 23.3%) was higher than *Anaplasma* spp. (174/800, 21.7%). Amplified partial 18S rRNA genes were sequenced and enrolled animals were found to be infected by *T. ovis* (115/800, 14.3%), and at least two more *Theileria* species (72/800, 9%) were present (*T. lestoquardi* and *T. annulata*). All blood samples that were found to be positive for *Anaplasma* spp. were also positive for *A. ovis*. Infection prevalence was higher in sheep (227/361, 28.3%) compared to goats (134/361, 16.6%) (*p* < 0.005). Univariable analysis of risk factors showed that host, age, grazing system and acaricide treatment were significant determinants (*p* < 0.05) for both *Theileria* and *Anaplasma* infections. Multivariable analysis revealed that host, sex, age, tick infestation and grazing system were significant risk factors (*p* < 0.005) for both pathogens. Phylogenetic analysis revealed variants among the *A. ovis* and *T. annulata* samples analysed, indicating that different genotypes are circulating in the field while *T. ovis* presented the same genotype for the samples analysed.

## Abstract (fr)

Cette étude a été menée dans quatre districts (Malakand, Swat, Bajaur et Shangla) du nord du Pakistan pour étudier la prévalence, les facteurs de risque associés et les analyses phylogénétiques des espèces de *Theileria* et *Anaplasma* chez les petits ruminants. Au total, 800 échantillons de sang, 200 de chaque district, ont été prélevés sur des animaux apparemment sains. Les tests PCR ont été réalisés en utilisant des amorces génériques pour *Anaplasma* spp. et *Theileria* spp. ainsi que des amorces spécifiques à l’espèce pour *A. ovis* et *T. ovis*. La prévalence globale de l’infection était de 361/800 (45,1 %). La prévalence de l’infection à *Theileria* spp. (187/800, 23,3 %) était plus élevée que celle d’*Anaplasma* spp. (174/800, 21,7 %). Le gène de l’ARNr partiel 18S amplifié a été séquencé et les animaux concernés se sont révélés infectés par *T. ovis* (115/800, 14,3 %) et au moins deux autres espèces de *Theileria* (72/800, 9 %) étaient présentes (*T. lestoquardi* et *T. annulata*). Tous les échantillons de sang trouvés positifs pour *Anaplasma* spp. ont également été trouvés positifs pour *A. ovis*. La prévalence de l’infection était plus élevée chez les moutons (227/361, 28,3 %) que chez les chèvres (134/361, 16,6 %) (*p* < 0,005). Une analyse univariée des facteurs de risque a montré que l’hôte, l’âge, le système de pâturage et le traitement acaricide étaient des déterminants significatifs (*p* < 0,05) pour les infections à *Theileria* et *Anaplasma*. L’analyse multivariée des facteurs de risque a révélé que l’hôte, le sexe, l’âge, l’infestation par les tiques et le système de pâturage étaient des éléments de facteurs de risque importants (*p* < 0,005) pour les deux agents pathogènes. L’analyse phylogénétique a révélé des variantes parmi les échantillons d’*A. ovis* et de *T. annulata* analysés indiquant que différents génotypes circulent sur le terrain tandis que *T. ovis* présentait le même génotype pour tous les échantillons analysés.

## Introduction

Sheep and goats play a key role in the economy of Pakistan [66]. This is shown by the fact that Pakistan has the third largest goat population (76.1 million) and the 12th largest sheep population (30.9 million) in the world [27]. The Pakistani livestock sector contributed 11.2% to the gross domestic product (GDP) in 2018–2019, and a total of 48.8 thousand tons of meat and its products were exported during this period, generating income of US$ 198.8 million and representing approximately 3.1% of total national exports. It is estimated that livestock production is the source of 35–40% of income for over 8 million rural families in Pakistan [41]. The Punjab and Sindh provinces of Pakistan have suitable land for crops and have more developed industrial sectors, but the population of Khyber Pakhtunkhwa (KP) province is highly dependent on livestock production and it significant contributes to livelihoods [13, 39]. Successful livestock production is hampered by a variety of factors such as mismanagement, infestation by ectoparasites, contagious infectious diseases, and nutritional deficiencies, preventing KP from fulfilling the meat, milk and hide requirements for an increasing human population [58]. One main cause of this inefficiency is tick-borne diseases [23]. The most important tick-borne diseases reported from tropical and sub-tropical areas are theileriosis and anaplasmosis, caused by intraerythrocytic pathogens of the genera *Theileria* and *Anaplasma*, respectively [19, 45]. There are six *Theileria* species that cause ovine theileriosis in small ruminants: *Theileria* (*T.*) *ovis*, *T. separata*, *T. uilenbergi*, *T. recondita*, *T. lestoquardi*, and *T. luwenshuni* [34, 47, 52, 61, 63]. Among the *Theileria* spp., *T. ovis*, *T. recondita* and *T. separata* have low pathogenicity [8]. *Theileria luwenshuni* and *T. uilenbergi* are considered more pathogenic, while *T. lestoquardi*, causing malignant theileriosis, is the most pathogenic among all small ruminant *Theileria* species [51]. The clinical signs of theileriosis consist of fever, nasal discharge, jaundice, anaemia, lacrimation, enlargement of superficial lymph nodes, rapid weight loss, anorexia, reduced appetite and anaemia [39, 57]. *Anaplasma* species that are considered to infect small ruminants are *A. marginale*, *A. ovis*, and *A. phagocytophilum*, and a recently identified *A. capra* [46, 48]. *Anaplasma capra* is a zoonotic species detected in humans and sheep, and goats, cattle and wild ruminants are considered to be reservoirs [40]. *Anaplasma phagocytophilum* is the causative agent of granulocytic anaplasmosis, which infects the neutrophils of humans and causes tick-borne fever in small ruminants, dogs and horses [62]. Of these *Anaplasma* spp., *A. marginale* and *A. ovis* infect small as well as large ruminants worldwide and have considerable economic importance [30, 36]. *Anaplasma ovis* is moderately pathogenic in sheep, goats and wild ruminants [53]. *Anaplasma marginale* is related to acute anaplasmosis characterized by pale mucous, weight loss, severe anaemia, pyrexia, jaundice, decreased milk production, nervous signs, constipation, dehydration, loss of wool, coughing, laboured breathing, abortion, and sometimes death of the infected animal [1]. Ticks of *Hyalomma* spp. and *Haemaphysalis* spp. are known to transmit *Theileria* spp. to small ruminants [9] while anaplasmosis is biologically transmitted to ruminants by about 20 species of ixodid ticks including *Rhipicephalus* spp., *Hyalomma* spp. and *Dermacentor* spp. Among these, *Rhipicephalus microplus* is globally reported to be the major *Anaplasma* spp. vector tick [30]. Mechanical transmission of *Anaplasma* spp. by the genera *Tabanus*, *Stomoxys*, and *Psorophora* or through contaminated fomites is frequent [11, 31, 48].

Various risk factors such as the sex of the animal, herd management, seasons, tick presence, and herd size are reported to be associated with theileriosis and anaplasmosis. Spatio-temporal conditions such as vector habitat, bacterial populations, animal grazing systems, hygiene and management practices also affect the epidemiology of the infection [32]. The aim of this study was to estimate the prevalence of *Theileria* spp. and *Anaplasma* spp. in sheep and goat blood samples collected from four districts in northern Pakistan and to report the risk factors that are associated with the prevalence of these pathogens.

## Materials and methods

### Ethics approval

The study design and experimental protocols were approved by the ethics committee of the Department of Zoology, Abdul Wali Khan University Mardan, Pakistan (approval number Awkum-Z00-1125). Written and informed consent was obtained from veterinary hospitals, from where samples were collected, and from livestock owners.

### Study area and number of samples

**Figure 1:**
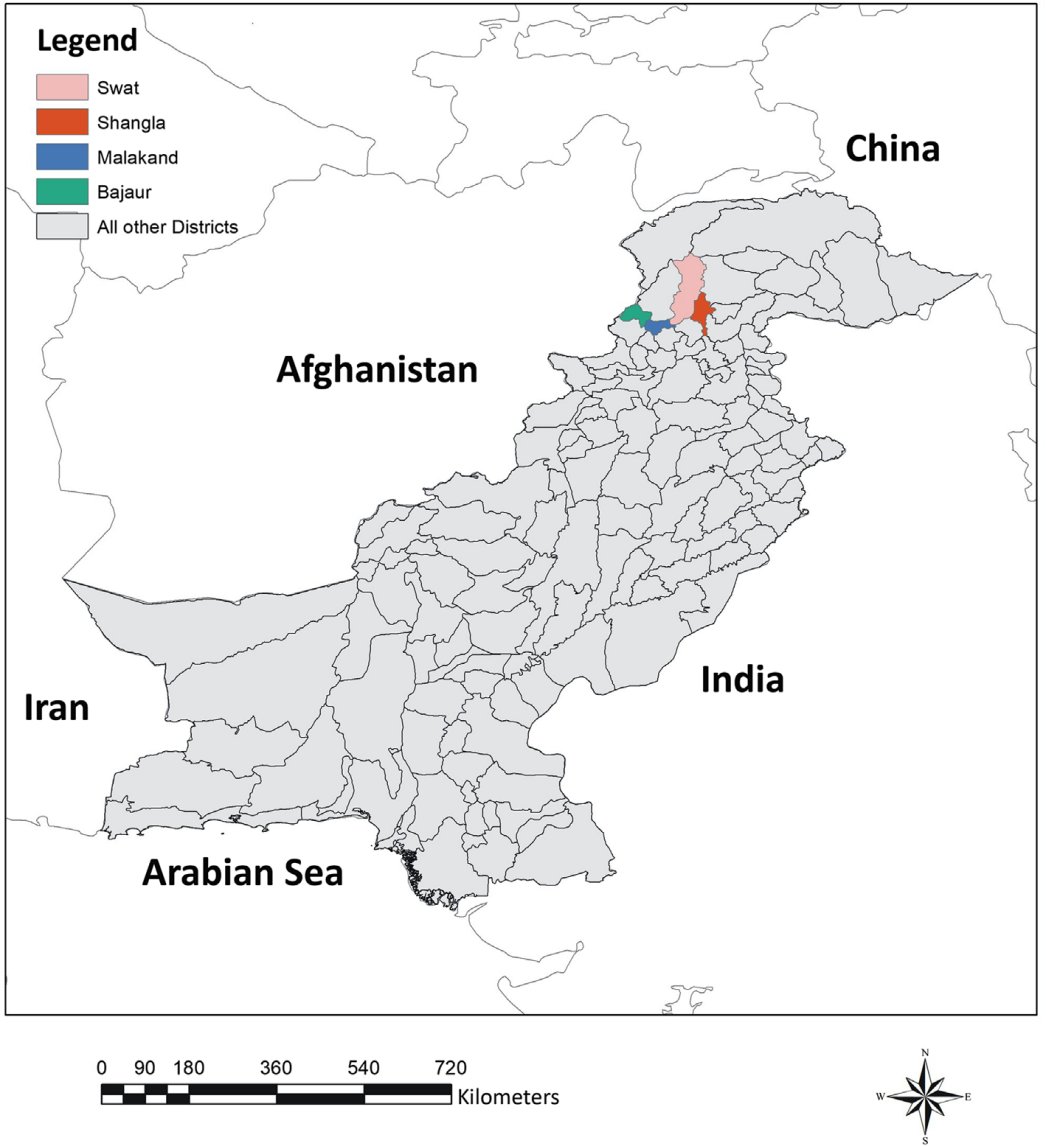
Map showing geographic location of study area (Northern Pakistan).

A total number of 800 blood samples were collected between January and December 2019 from four districts (Malakand [34°30′10.8″ N, 71°54′16.56″ E], Swat [35°13′21.72″ N, 72°25′32.88″ E], Bajaur [34°47′11.4″ N, 71°32′5.64″ E] and Shangla [34°53′13.92″ N, 72°45′25.2″ E]) of northern Pakistan (Fig. 1). A random sampling approach was applied to collect blood samples from symptomatic and asymptomatic goats (*n* = 401) and sheep (*n* = 399). From each district, 200 blood samples were collected, and enrolled animals included 281 males and 519 females. In these districts, livestock are managed on pastures as well as stall feeding under a traditional management system. These areas are sub-tropical dry mixed deciduous scrub forest and dry subtropical temperate semi-evergreen scrub forest. The annual average temperature of the studied areas ranges from 20 to 40 °C during the hottest months of the year with 65% average humidity [49].

### Demographic characteristic of the study population

A total of 800 goats and sheep were studied, among which almost equal numbers of goats 401 (50.1%) and sheep 399 (49.9%), with animals < 6 months and animals ≥ 6 months of age also included with the same ratio of 50.1% and 49.9%, respectively. Sex-based sampling analysis indicated that the majority of the enrolled animals were female (*n* = 519; 64.9%) goats and sheep as compared to males (*n* = 281; 35.1%). The majority of the sampled animals were free-grazing (*n* = 300; 37.5%) followed by semi-grazing (*n* = 196; 24.5%) and zero-grazing animals (animals with stall feeders restricted to their homes) (*n* = 304; 38%). A total of 265 (33.1%) goats and sheep were treated regularly, 200 (25%) were irregularly treated with acaricides and 335 (41.9%) never received any acaricidal treatment. About 452 (65.5%) of the investigated population was not infected by ticks (Table 1).

**Table 1:** Demographic properties of the study population from study area.

Variables	Categories	Shangla	Swat	Malakand	Bajaur	Total (%)
Age	Young	70	74	69	71	401 (50.1)
	Adult	130	126	131	129	399 (49.9)
Sex	Male	75	67	64	75	281 (35.1)
	Female	125	133	136	125	519 (64.9)
Host	Goat	101	100	100	100	401 (50.1)
	Sheep	99	100	100	100	399 (49.9)
Ticks	Present	74	74	94	106	348 (43.5)
	Absent	126	126	106	94	452 (65.5)
Grazing	Free	85	87	37	91	300 (37.5)
	Semi	48	48	52	48	196 (24.5)
	Zero	67	65	111	61	304 (38.0)
Acaricides	Regular	68	70	57	70	265 (33.1)
	Irregular	33	54	76	37	200 (25.0)
	No use	99	76	67	93	335 (41.9)

### Risk factors analysis

A questionnaire was designed to gather data regarding the risk factors (age, sex, host type (goat or sheep), acaricide applications, grazing system, tick infestation) associated with the studied pathogens, and it was completed on the spot at the time of blood sampling. Goats and sheep were categorised into two age groups: <6 months and ≥6 months of age. The whole body of each animal was thoroughly inspected for tick infestations.

### Blood sample collection

Blood samples were collected in EDTA tubes (Thermo Fischer Scientific™, USA) and kept at +4 °C until they were further processed at the parasitology laboratory of the College of Veterinary Sciences and Animal Husbandry, Abdul Wali Khan University Mardan, Pakistan.

### Molecular detection of *Theileria* and *Anaplasma* species

DNA was extracted from the preserved blood samples with a Gene all Kit (Exgene™ Blood SV, Germany), according to the manufacturer’s instructions. DNA was quantified using a NanoDrop™ 1000 spectrophotometer (NanoDrop Technologies Inc., Wilmington, DE, USA) based on 260/280 nm absorbance ratios, and then stored at −80 °C for future analyses.

PCR-based assays were used to screen the collected samples. Generic primers that amplify the 16S small subunit ribosomal RNA (SSU rRNA) of *Anaplasma* spp. (577 bp) [45] and 18S SSU rRNA gene of *Theileria* spp. (1098 bp) were used [4]. Species-specific primers were used to amplify an 870 bp [46] fragment from the *msp4* gene of *A. ovis* and a 520 bp fragment from the small subunit ribosomal RNA gene of *T. ovis* [5]. PCR assays were performed with 250 ng of genomic DNA using a MasterMix (including Taq polymerase, dNTP (0.4 mM each) and buffer with 2 mM MgCl_2_; Thermo Fisher Scientific) and distilled water in a final volume of 25 μL. DNA samples positive for *Theileria* species, *T. ovis*, *Anaplasma* species and *A. ovis* were kindly provided by Prof. Dr. Munir Aktas at Firat University, Turkey and DNA of *T. lestoquardi* was provided by the Immunology and Cell Biology Research Center, Borstel, Germany and they were used as positive controls. Deionised water was used as negative control.

The thermoprofile that was used to detect *Anaplasma* spp., and *Theileria* spp*.* consisted of: denaturation for 7 min at 95 °C followed by 30 cycles (denaturation at 95 °C for 30 s, annealing at 58.3 °C for 45 s and extension for 30 s at 72 °C), followed by a final extension at 72 °C for 10 min. Cycling conditions for the species-specific primers for the detection of *T. ovis* and *A. ovis* were the same as above, except that the number of cycles was increased to 35 and annealing temperature was 60 °C for 1 min. All PCR products were checked in 1.5% agarose gel stained with 0.5 μg/mL ethidium bromide and visualised under UV light (Bio-Rad laboratories, CA, USA).

### DNA sequencing and phylogenetic analyses

PCR products were chosen, cleaned and sent to a commercial company (Macrogen, Inc., Seoul, South Korea) for Sanger sequencing in both directions (3′ – 5′ and 5′ – 3′) of the SSU rRNA gene of *Theileria ovis* and *Theileria* spp., and the *msp4* gene of *Anaplasma ovis*.

Newly obtained sequences were compared with the NCBI database using the Basic Local Alignment Search Tool (BLAST) (https://blast.ncbi.nlm.nih.gov/Blast.cgi) and related sequences were obtained from the GenBank database (https://www.ncbi.nlm.nih.gov/), with multiple alignments among sequences carried out with MUSCLE [14]. Phylogenetic analyses were conducted in MEGA, version X [33] using the top-ranking substitution models according to lowest BIC scores (Bayesian Information Criterion) and a bootstrap analysis with 1000 replicates. Phylogenetic trees were visualised and edited with iTOL (https://itol.embl.de/). Phylogenetic analyses of the *msp4* gene from *A. ovis* and the SSU rRNA gene from *T. ovis*, were performed using the UPGMA method [56] with the Kimura 2-parameter model [29] and the Jukes-Cantor model [25], respectively. For SSU rRNA from *T. annulata* and *T. lestoquardi*, evolutionary histories were inferred using the Maximum Likelihood method with Kimura 2-parameter model [29]. Datasets of sequences obtained from GenBank and those generated in this study were trimmed to 854, 1056 and 482 bp for *msp4* from *A. ovis*, SSU rRNA from *T. annulata* and *T. lestoquardi*, and SSU rRNA from *T. ovis*, respectively. Identity scores between the sequences were based on multiple alignments and calculated with the SIAS tool (http://imed.med.ucm.es/Tools/sias.html).

### Statistical analyses

All statistical analyses for the present study were carried out using R software, version 3.5.1 [43]. A chi-square test was used to compare prevalence between districts. For the determination of statistical significance and association between the pathogen prevalence and other independent variables, univariable and multivariable regression models (Generalized linear model univariable/Mix multivariable model) were used.

## Results

### PCR-based prevalence of *Theileria* spp. and *Anaplasma* spp.

Overall, 361/800 (45.1%) animals were found to be infected by one of the haemopathogens, among them, 187/800 (23.37%) were infected by *Theileria* spp., while 174/800 (21.75%) were infected with *Anaplasma* spp. Prevalence of *Theileria* spp. and *Anaplasma* spp. coinfection was higher in sheep (14.5 and 13.87%, respectively) than in goats (8.87 and 7.87%, respectively) (*p* < 0.001) (Tables 2 and 3).

**Table 2:** Prevalence rates of *Theileria* spp*.*, *Theileria ovis*, and *Anaplasma ovis* in small ruminants from four districts in Khyber Pakhtunkhwa. The *p*-value represents the results of Chi-square tests conducted to report the prevalence of a parasite among the sampling districts.

Districts	*Theileria* spp.						*Anaplasma* spp*.*			Total% (CI)
	*T. ovis n* (%)	CI 95%	*p*-value	*Theileria* spp. n (%)	CI 95%	*p*-value	*A. ovis* n (%)	CI 95%	*p*-value	
Shangla	21/200 (10.5)	6.62–15.60	0.2	14/200 (7)	3.88–11.47	0.7	32/200 (16.0)	11.21–21.83	0.08	33.5 (27.00–40.50)
Swat	32/200 (16.0)	11.21–21.83		19/200 (9.5)	5.82–11.44		48/200 (24.0)	18.26–30.53		49.5 (42.37–56.64)
Malakand	35/200 (17.5)	12.50–23.49		21/200 (10.5)	6.62–15.60		52/200 (26.0)	20.07–32.66		54.0 (46.83–61.05)
Bajaur	27/200 (13.5)	9.09–19.03		18/200 (9)	5.42–13.85		42/200 (21.0)	15.57–27.31		43.5 (36.52–50.67)
Total	115/800 (14.3)			72/800 (9)			174/800 (21.7)			361/800 (45.1)

CI = Confidence interval.

**Table 3:** Results of univariable and multivariable logistic regression analysis of *Theileria* spp*.* and *Anaplasma ovis* PCR-positive infections in sheep and goats in Northern Pakistan.

Risk factors	*Theileria* spp*.*						*Anaplasma ovis*					
	Prevalence		Univariable		Multivariable		Prevalence		Univariable		Multivariable	
	Positive (%)	CI 95%	OR (95% CI)	*p*-value	OR (95% CI)	*p*-value	Positive (%)	CI 95%	OR (95% CI)	*p*-value	OR (95% CI)	*p*-value
Host												
Goat	71 (8.8)	6.83–10.76	0.52 (0.37–0.73)	0.001	0.42 (0.27–0.65)	0.001	63 (7.8)	6.01–9.74	0.48 (0.34–0.68)	0.001	0.83 (0.22–3.86)	0.001
Sheep	116 (14.5)	12.06–16.33					111 (13.8)					
Sex												
Male	59 (7.3)	5.55–9.18	0.8 (0.56–1.14)	0.242	0.54 (0.33–0.85)	0.001	56 (7.0)	5.23–8.76	0.84 (0.58–1.20)	0.35	0.61 (0.23–2.63)	0.001
Female	128 (16.0)	13.45–18.54					118 (14.7)	12.29–17.20				
Age												
< 6 months	114 (14.2)	11.71–16.53	3.7 (2.71–5.36)	0.001	3.77 (2.44–5.87)	0.001	100 (12.5)	10.20–14.79	3.24 (2.30–4.60)	0.001	1.33 (0.22–5.88)	0.001
≥ 6 months	73 (9.1)	7.24–11.25					74 (9.2)	7.24–11.25				
Tick infestation												
Present	174 (21.7)	18.89–24.61	3.43 (0.29–11.75)	0.001	20.9 (11.01–42.63)	0.001	162 (20.2)	17.46–23.03	3.46 (0.31–11.11)	0.001	3.04 (0.34–8.85)	0.001
Absent	14 (1.7)	0.84–2.65					12 (1.5)	0.65–2.35				
Grazing system												
Full grazing	125 (15.6)	13.11–18.14					116 (14.5)	12.06–16.93				
Semi	40 (5)	3.48–6.51	0.32 (0.26–0.41)	0.001	0.57 (0.42–0.75)	0.001	41 (5.1)	3.57–6.62	0.34 (0.26–0.42)	0.001	0.56 (0.14–3.88)	0.001
Zero	21 (2.6)	1.51–3.73					17 (2.1)	1.10–3.09				
Acaricides												
Regular	13 (1.6)	0.73–2.46					12 (1.5)	1.41–3.58				
Irregular	40 (5.0)	3.48–6.51	3.30 (2.58–4.29)	0.001	1.25 (0.88–1.01)	0.18	36 (4.5)	3.06–5.93	3.30 (2.57–4.33)	0.001	0.22 (0.17–1.29)	0.19
No use	134 (16.7)	14.16–19.33					126 (15.7)	13.17–18.22				

OR = Odds ratio, CI = Confidence interval. Semi = semi-grazing system.

The highest overall prevalence of *Theileria* spp*.* and *Anaplasma* spp. was observed in the Malakand district 108/200 (54%) followed by Swat 99/200 (49.5%), Bajaur 87/200 (43.5%) and Shangla 67/200 (33.5%). Prevalence of pathogens varied non significantly (*p* > 0.05) when compared between the sampling districts (Table 2). All 187 samples positive for *Theileria* spp. (23.3%) were analysed for *Theileria ovis*, and it was found that 14.3% (115/187) of animals (goats and sheep) were infected with *T. ovis* while 9% (72/187) were infected with other *Theileria* spp. (Table 2). Four *Theileria* spp.-positive samples were analysed by sequencing their 18 SSU rRNA gene and it was observed that at least two more *Theileria* species (*T. lestoquardi* and *T. annulata*) besides *T. ovis*, were present in these districts (Table 2, Fig. 4). All 174 blood samples that were found to be positive for *Anaplasma* spp. were also positive for *A. ovis* (21.7%) (Table 2).

Of the 200 animals analysed from each district, prevalence of *T. ovis* and *Theileria* spp*.* were highest in Malakand district (17.5% and 10.5%), followed by Swat district (16% and 9.5%), Bajaur district (13.5% and 9%) and Shangla district (10.5% and 7%). On the other hand, the highest prevalence of *A. ovis* was observed in Malakand district (26.0%), while lowest prevalence was recorded in Shangla district (16.0%) (Table 2).

### *Anaplasma* spp. risk factor analysis

When risk factor data were analysed, it was observed that prevalence of *Anaplasma* spp. infection was higher in young animals <6 months than in older animals ≥6 months of age: 100/800 (12.5%) vs. 74/800 (9.2%), respectively (*p* < 0.001). Based on different types of hosts, *Anaplasma* spp. was more common in sheep (111/800; 13.8%) than in goats (63/800; 7.8%) (*p* < 0.001). *Anaplasma* spp. 126/800 (15.7%) prevalence was higher in animals that were not treated with acaricides, followed by irregularly treated animals (36/800; 4.5%), while the lowest prevalence (12/800; 1.5%) was found in animals that were regularly treated with acaricides (*p* < 0.001). Grazing practices showed that the prevalence of *Anaplasma* spp. was higher (116/800; 14.5%) in full-grazing practices, than irregular practices (41/800; 5.1%), while the zero-grazing animals were least infected with *Anaplasma* spp. (17/800; 2.1%) (*p* < 0.001). *Anaplasma* spp. was highly prevalent in tick-infested goats and sheep, with a prevalence rate of 162/800 (20.2%). However, goats and sheep not infested with ticks (12/800; 1.5%) were found to be infected with *Anaplasma* spp.

### Univariable and multivariable risk factors regression analyses of *Anaplasma* spp. infection

The potential risk factors associated with *Anaplasma* spp. infection based on univariable regression analyses were host (OR = 0.48, CI = 0.34–0.68, *p* = 0.001), age (OR = 3.24, CI = 2.30–4.60, *p* = 0.001), tick infestation (OR = 3.46, CI = 0.31–11.11, *p* = 0.001), grazing system (OR = 0.34, CI = 0.26–0.42, *p* = 0.001) and acaricide use (OR = 3.30, CI = 2.57–4.33, *p* = 0.001). Similarly, the same factors (host, age, tick infestation, grazing system and acaricide use) were also statistically significant in the multivariable regression analyses with ORs of 0.83, 1.33, 3.04, 0.56. and 0.22, along with *p* = 0.001. Sex was only an associated potential risk factor on the multivariable regression analyses (OR = 0.61 CI = 0.23–2.63, *p* = 0.001); *Anaplasma* spp. was more prevalent in females (118/800; 14.7%) than males (56/800; 7.0%).

### *Theileria* spp. risk factor analysis

The risk factor analysis for *Theileria* spp. showed that young animals (<6 months) had a higher infection rate (114/800; 14.2%) than adult animals ≥ 6 months of age (73/800; 9.1%) (*p* < 0.001), and the rate was higher in sheep (116/800; 14.5%) than in goats (71/800; 8.8%) (*p* < 0.001) (Table 3). *Theileria* prevalence (134/800; 16.7%) was higher in animals with no acaricide treatment, followed by irregularly treated animals (40/800; 5%), and animals that were regularly treated with acaricides presented the lowest prevalence (13/800; 1.6%) (*p* < 0.001). Animals in full-grazing systems had higher prevalence (125/800; 15.6%) than those in a semi-grazing (40/800; 5%) or zero-grazing systems (13/800; 2.6%) (*p* < 0.001). Animals infested with ticks had higher prevalence (174/800; 21.7%) compared with animals with no ticks (14/800; 1.7%) (*p* < 0.001).

### Univariable and multivariable regression analyses for *Theileria* spp. infection risk factors

The potential risk factors associated with *Theileria* spp. infection based on univariable regression analyses were host (OR = 0.52, CI = 0.37–0.73, *p* = 0.001), age (OR = 3.7, CI = 2.71–5.36, *p* = 0.001), grazing system (OR = 0.32, CI = 0.26–0.41, *p* = 0.001), tick infestation (OR = 3.43, CI = 0.29–11.75, *p* = 0.001) and acaricide use (OR = 3.30, CI = 2.58–4.29, *p* = 0.001). The same risk factors such as host (OR = 0.42, CI = 0.27–0.65, *p* = 0.001, age (OR = 3.77, CI = 2.44–5.87, *p* = 0.001), tick infestation (OR = 20.9, CI = 11.01–42.63, *p* = 0.001), and grazing system (OR = 0.32, CI = 0.26–0.41, *p* = 0.001) were associated with *Theileria* spp. infection in the multivariable regression analyses. Sex was associated as a potential risk factor only on the multivariable regression analyses (OR = 0.54, CI = 0.33–0.85, *p* = 0.001), with a prevalence of 128/800 (16%) in females and 59/800 (7.3%) in males.

### Phylogenetic analysis

**Figure 2:**
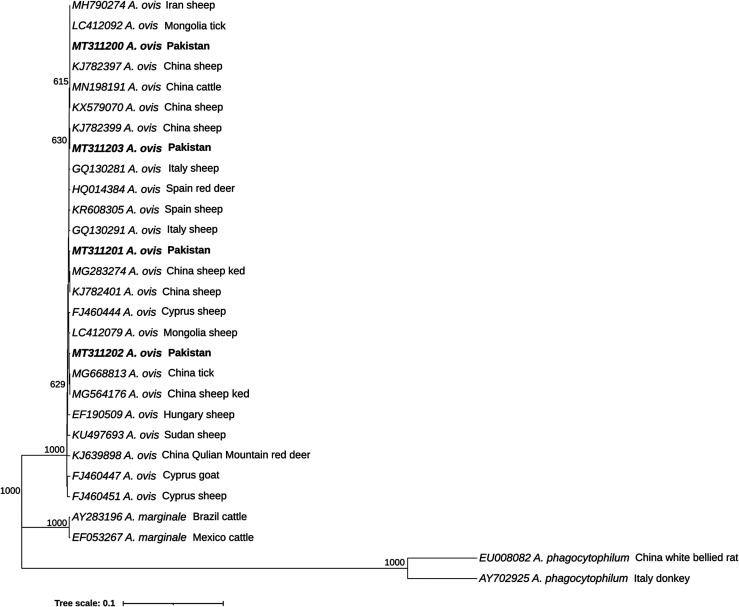
Phylogenetic analysis of Anaplasma ovis based on the msp4 gene. The sequences determined in this study are shown in bold font. Numbers at the nodes represent the number of occurrences of clades in 1000 bootstrap replications of the taxa. Phylogenetic analysis was performed using MEGA 10 software. The *msp4* gene sequences of *Anaplasma marginale* (EF052297 and AY283196) and *Anaplasma phagocytophilum* (EU008082 and AY702925) were used as the outgroup.

The *msp4* gene sequences from *A. ovis* were deposited in GenBank under accession numbers MT311200, MT311201, MT311202, and MT311203. The small subunit ribosomal RNA gene sequences from *T. ovis* were deposited under accession numbers MT318208, MT318209, and MT318210, from *T. lestoquardi* under accession number MT318171, and from *T. annulata* under accessions numbers MT318158, MT318159 and MT318158. *Anaplasma ovis* from Pakistan clearly clustered with other *A. ovis* sequences (Fig. 2); however, based on a multiple alignment (data not shown), the sequences present nucleotide differences between them, suggesting that different strains are circulating in the analysed areas.

**Figure 3:**
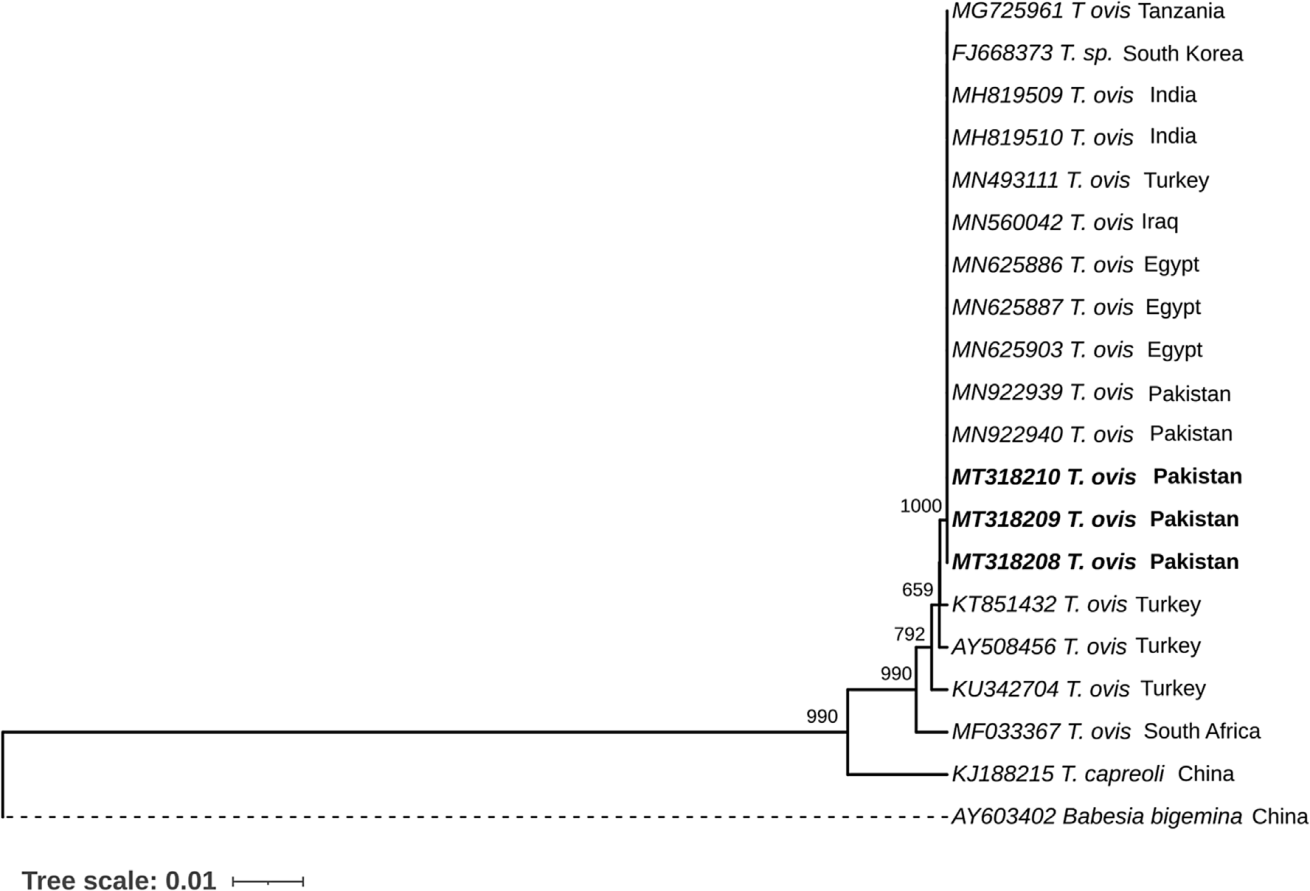
Phylogenetic analysis of Theileria ovis based on the SSU rRNA gene. The sequences generated in this study are shown in bold font. Numbers at the nodes represent the number of occurrences of clades in 1000 bootstrap replications of the taxa. Phylogenetic analysis was performed using MEGA 10 software. The SSU rRNA gene sequences of *Babesia bigemina* (AY603402) and *Theileria capreoli* (KJ188215) were used as the outgroup.

Concerning the *T. ovis* PCR samples that were amplified with the specific primers for *T. ovis*, three sequences were phylogenetically analysed to reveal the relationship between SSU rRNA gene sequences and other *Theileria* species sequences obtained from GenBank. According to the tree, sequences MT318208, MT318209 and MT318210 distinctly clustered with *T. ovis* sequences (Fig. 3) and they shared 100% identity between them and with another *T. ovis* strain isolated from Pakistan (accession numbers: MN922940 and MN922939), Egypt (accession numbers: MN625903 and MN625887), Iraq (accession number: MN560042), Turkey (accession number: MN493111), India (accession numbers: MH819509 and MH819510), Tanzania (accession number: MG725961), and South Korea (accession number: FJ668373) based on a multiple alignment (data not shown).

**Figure 4:**
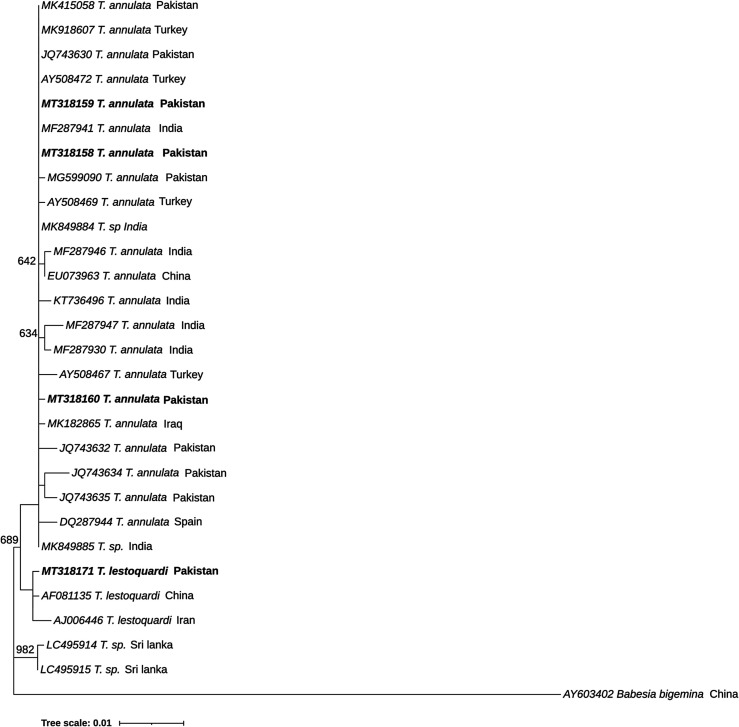
Phylogenetic analysis of *Theileria annulata* and *Theileria lestoquardi* based on the SSU rRNA gene. The sequences in bold font are from this study. The numbers at the nodes represent the number of occurrences of the clades in 1000 bootstrap replications of the taxa. Phylogenetic analysis was performed using MEGA 10 software. The SSU rRNA gene sequence of *Babesia bigemina* (AY603402) was used as the outgroup.

Four DNA sequences that were found to be positive following their amplification with generic primers for *Theileria* spp. were phylogenetically analysed to reveal the relationship between their SSU rRNA gene sequences and other *Theileria* species sequences obtained from GenBank. One of these sequences (MT318171) clustered with *T. lestoquardi* sequences. The other three sequences (MT318158, MT318159 and MT318160) clustered with *T. annulata* sequences (Fig. 4).

## Discussion

To our knowledge, this is the first study conducted for molecular detection and phylogenetic analysis of tick-borne pathogens in small ruminants from four different districts of Khyber Pakhtunkhwa, northern Pakistan (Malakand, Swat, Bajaur and Shangla). This study determined the status of tick-borne diseases in small ruminants from these areas that were affected by war for more than 20 years. The geographical location of the study area is very important as its boundaries are aligned with two neighbouring countries, Iran and China.

Three *Theileria* spp. (*T. ovis*, *T. lestoquardi* and *T. annulata*) and one *Anaplasma* sp. (*A. ovis*) of haemopathogens were identified both in goats and sheep.

### Prevalence

The study revealed an overall prevalence of 45.1% for both pathogens in small ruminants from northern Pakistan of which 23.3% were *Theileria*, which is consistent with previous studies (17.4–31.2%) from Bannu, Tank and Dera Ismail Khan districts in southern Khyber Pakhtunkhwa [58] and elsewhere (17.8 and 17.2% in Turkey and China, respectively) [8, 64]. However, some studies reported contrasting prevalences; for example, a study by Nasreen et al. [38] in the Dir District, Lower KP showed a prevalence of 53.5%, while Durrani et al. [13] reported a prevalence of 35% from Lahore with PCR. Similarly, a lower prevalence (3.47%) was reported in Southern Punjab [17], 6% in Kohat and Peshawar [13], while Saeed et al. [50] reported a prevalence of 3% in Kohat and Peshawar. These differences may be due to different geographical areas and climatic conditions [13]. In our study, *T. ovis* was more prevalent than *Theileria* spp*.* which is consistent with previously studies from different parts of the country [38, 47] and elsewhere [61]. *Theileria annulata* is mainly a pathogen of cattle; experimental infections of sheep and goats with *T. annulata* cause mild symptoms with no development of piroplasm [35]. It is necessary to study the clinical signs of *T. annulata* on naturally infected animals to determine the clinic importance of this infections and the role of animals as a reservoir for the pathogen in the area.

Similarly, our results showed an overall prevalence of 21.7% for *Anaplasma* spp. which is similar to previous findings in Mardan District, Khyber Pakhtunkhwa, Pakistan with 23.33% using cELISA [37]. However, the prevalence we estimated was slightly lower than those previously reported by Hussein et al [22] (56.25%) and by Shah et al. [55] (40.7%) in Karak and Peshawar, respectively.

The overall prevalence of *A. ovis* was estimated to be 21.7%, which falls within the range of 11.5–87.4% previously reported across the world by other authors [10, 12, 24, 36, 46, 54].

The PCR results revealed in sheep and goats an *Anaplasma ovis* prevalence rate of 16.0, 21.0, 24.0 and 26.0% for Shangla, Bajuar, Swat and Malakand districts, respectively. These prevalences are lower than those reported from Turkey (71.32%) and Pakistan (56.25%) [6, 22], but higher than those reported in China (11.7%) and the United States (14%) in sheep [18, 42]. Similarly, higher molecular *Anaplasma* prevalences have been reported in Iranian goats (38.9%) [44] and (63.7%) [2]. These variations may be due to geographical location, different diagnostic methods used, animal breeds with different susceptibility to pathogens, proximity of veterinarian services, seasons of sample collection, climatic differences, tick infestation intensity, and absence of mechanical and biological vectors in that particular area.

In the current study, the prevalence of *Theileria* and *Anaplasma* was significantly higher in sheep (14.5 and 13.9%) than in goats (8.8 and 7.9%), which may be due to the nature of their skin. The skin of goats is thinner and shows resistance to the attachment of ticks due to its smooth aspect, while sheep have wool and ticks become easily entangled making sheep more susceptible to infection [21, 47, 54]. The lower prevalence in goats than in sheep could also be due to the ability of goats to pasture in areas that are steep and poorly accessible, where there are far fewer opportunities for contact with ticks that feed on other animals during their life cycle [3].

Our results showed that animals younger than 6 months of age had higher levels of theileriosis infection. This may be due to an underdeveloped immune system, deficient grooming, or another risk factor that was not analysed in this study, such as vaccination or weaning. It has previously been reported that goats 3–6 months of age had a higher risk of infection than other age groups, with a reported infection rate of 19.2% (95% CI: 16.4–22.0%) [60].

In the present study, a higher prevalence of *Anaplasma* sp. was observed in young animals compared to adults; similar findings were also reported previously [20, 54]. Similarly, sex-specific data revealed that females showed a higher prevalence compared to males, findings consistent with those previously reported from different regions of Pakistan [7, 13, 65] and Nigeria [26]. Differences may be due to several factors, including the fact that females are frequently under stress: pregnancy and lactation [7].

### Risk factors

The risk factors of host, age and grazing system were statistically significant for *Theileria* infection by both univariate and multivariate analysis. Grazing animals are at a higher risk than non-grazing animals as a result of greater exposure to tick infestation since they are exophilic [15]. Interestingly, acaricide application was statistically significant in the case of univariable analysis; however, its effect was non-significant in the multivariable analysis. Similar results were reported by Nasreen et al [38]. Risk factors such as host, age and grazing system were statistically significant for *Anaplasma* in both multivariable and univariable analysis. Obviously, animals infested with larger numbers of ticks are at greater risk of both *Anaplasma* and *Theileria* infections [17, 59].

### Phylogenetic analyses

Phylogenetic analyses revealed that *Anaplasma ovis* detected in the four different districts are genetically different, indicating that different genotypes are circulating in these regions. The sequences shared identity scores of 99.6%–99.9% between themselves and 99%–100% with already reported *msp4* sequences of *A. ovis* identified in several host species from nine countries and that were used in this study. Genetic variability has also been reported for *A. ovis* using this marker [16, 36]. Isolate MT311200 from Pakistan was identical to sequences from China (accession numbers: K579070, MN198191 and KJ782397), Iran (accession number: MH790274) and Mongolia (accession number: LC412092). Sequence MT311203 was identical to an isolate from China (accession number: KJ782399), while MT311201 and MT311202 were distinct variants. The results of this study highlight the need for detailed investigations to characterise the regional genetic diversity of *A. ovis* in Pakistan and correlate it to virulence.

Three *Theileria* species were identified in the isolates analysed, namely, *T*. *ovis*, *T. annulata* and *T. lestoquardi*. No variation was found between the SSU rRNA gene sequences of *T. ovis* from this study and the *T. ovis* obtain from Africa (accession number: MG725961) and Asia (accession numbers: MH819510, MH819509, MN560042, MN493111, MN625903, MN625887 and MN625887). Furthermore, as shown in the phylogenetic tree, MT318208, MT318209 and MT318210 *T. ovis* SSU rRNA sequences also clustered with *T. ovis* sequences from Pakistan (accession numbers: MN922939 and MN922940) deposited in GenBank. Similar results have shown few or no variations between *T. ovis* isolates obtained from different countries, such as Kenya, Sudan and Tanzania, [36] suggesting that similar genotypes are circulating in several regions of the world.

In order to confirm and explore the sequence diversity of the *T. annulata* and *T. lestoquardi* sequences obtained in this study, a phylogenetic tree was constructed using the SSU rRNA gene. The *T. lestoquardi* sequence (accession number: MT318171) from this study clustered with *T. lestoquardi* sequences from China (accession number: AF081135) and Iran (accession number: AJ006446), in a separate clade. On the other hand, *T. annulata* showed sequence variants between the samples analysed in the present study. With respect to the *T. annulata* isolates from Pakistan and sequences reported herein, it was observed that the sequence with accession number MT318158 was identical to MT318159, while MT318160 shared an identity score of 99.90% with the other two sequences. *Theileria annulata* amplicons in the present study clustered with isolates of *T. annulata* from Pakistan, India, Spain, Turkey, Iraq and China, and they were clearly separated from the *T. lestoquardi* clade*. Theileria annulata* variants were previously reported from isolates from Pakistan districts along the India–Pakistan border [28]. These isolates also displayed variation from the isolates reported herein, highlighting the importance of addressing these variants in Pakistan in future studies. To evaluate the influence of legal and illegal animal trade and mobility, additional studies with larger sample sizes are required.

## Conclusion

This study demonstrated that *A. ovis*, *T. ovis*, *T. annulata* and *T. lestoquardi* are present in Northern Pakistan. *Theileria* spp. and *T. ovis* are more prevalent than *Anaplasma* spp*.* and these infections are more prevalent in sheep than in goats. Univariable analysis of risk factors showed that host, age, grazing system and acaricide treatment were significant risk factors (*p* < 0.05). Multivariable analysis of risk factors revealed that host, sex, age, tick infestation and grazing system were significant risk factors (*p* < 0.05) for both pathogens. Additionally, it was found that different genotypes of *A. ovis* and *T. annulata* are circulating in the field.

## Recommendation for future research

This study emphasises the need to conduct future research assessing the epidemiology of tick and tick-borne diseases across different agro-ecological zones, in different seasons and under various production systems to have a clear picture of the diseases and risk factors, which is very important to control these diseases in Pakistan.
